# LMRNet: a lightweight convolutional neural network for real-time mountain rice leaf disease recognition on edge devices

**DOI:** 10.3389/fpls.2026.1789523

**Published:** 2026-03-23

**Authors:** Jiapeng Cui, Yinyin Yang, Zhuo Chen, Shengqiang Hao, Feng Tan

**Affiliations:** 1 College of Mechanical Engineering, Chongqing Three Gorges University, Chongqing, China; 2 Chongqing Key Laboratory of Light Mountain Intelligent Agricultural Machinery, Chongqing Three Gorges University, Chongqing, China; 3 College of Information and Electrical Engineering, Heilongjiang Bayi Agricultural University, Daqing, China

**Keywords:** convolutional neural networks, disease Identification, edge intelligence, mountain rice, smart agriculture

## Abstract

**Introduction:**

Intelligent rice disease prevention and control are crucial components in the development of smart agriculture. In recent years, with the rapid advancement of computer vision technologies, a variety of deep learning-based methods for rice disease identification have been proposed, and some models have already surpassed the diagnostic performance of agricultural technicians. However, the existing models generally suffer from high computational complexity and limited generalization capabilities, rendering them difficult to deploy on edge devices for real-time and accurate disease recognition under offline field conditions.

**Methods:**

To promote engineering applications of related technologies, this study investigated leaf disease identification methods for mountain-grown rice oriented toward edge intelligence. Based on a self-constructed image dataset of mountain rice leaf diseases and following the design principles of lightweight convolutional neural networks, a novel lightweight mountain rice disease recognition model architecture suitable for edge intelligent devices was constructed. Furthermore, a mountain rice leaf disease recognition application was developed for smartphones on the Android platform.

**Results:**

Field validation experiments demonstrated that the application achieves an average accuracy of 92.41% across multiple disease categories and an average inference speed of approximately 22.47 frames per second on various smartphone models, indicating high real-time performance and recognition accuracy.

**Discussion:**

The research outcomes will provide a reliable theoretical foundation and technical support for the intelligent prevention and control of mountain rice diseases.

## 1 Introduction

Rice is one of the most important staple crops worldwide, and more than half of the global population relies on rice as a primary food source. Rice production is influenced by various environmental factors, such as light, temperature, and pathogenic microorganisms, with diseases being particularly prominent (Pandi et al., 2023). Rice pathogens can infect plants throughout the growing period, and severe infections can significantly reduce yield and grain quality, or even cause large-scale agricultural disasters. Therefore, real-time and accurate identification of rice diseases is crucial for ensuring food security (Sharma et al., 2023). Disease identification not only facilitates precise disease management and reduces yield losses but also helps prevent the excessive use of pesticides, thereby mitigating environmental pollution and aligning with the goals of green agriculture (Sharma et al., 2024). However, in many regions, particularly in developing countries, rice disease identification still relies largely on visual inspection and experiential judgment of skilled farmers or agricultural experts (Kalita et al., 2024). This traditional approach is constrained by the experience and availability of personnel and suffers from delays in diagnosis as well as high misidentification rates, rendering it difficult to meet the demands of modern agriculture for efficient and intelligent disease management (Nagpal and Goel, 2025). Therefore, a simple, rapid, low-cost, and accurate rice disease recognition system must be developed to enhance disease control efficiency and support the sustainable development of the rice industry.

In recent years, computer vision technology has developed rapidly owing to continuous advances in digital imaging and computing hardware performance. With advantages such as low cost, visualization, and noncontact operation, computer vision provides a new technical pathway for agricultural disease identification (Upadhyay et al., 2025). Consequently, computer vision-based approaches for rice disease recognition have emerged and have gradually become key research subject in this field. Existing studies can be broadly classified into three categories:

1. Rice disease identification based on digital image processing and traditional machine learning. Such methods typically involve four major steps: image preprocessing, lesion segmentation, feature extraction, dimensionality reduction, and classifier construction, all of which jointly determine the final recognition accuracy (Madhavan et al., 2021). Phadikar et al. (2013) constructed a dataset of 500 images containing four rice disease categories. They proposed an image segmentation approach based on Fermi energy, which effectively overcame the limitations of conventional thresholding, and employed rough set theory to extract the color, shape, and positional features of the lesion regions. Their customized classifier achieved a recognition accuracy of 94.21%. Shrivastava and Pradhan (2020), using only color features, extracted 172 color descriptors from a dataset of 619 rice disease images and compared seven classifiers. Their results indicated that the SVM achieved the best performance, with an accuracy of 94.65%. Kishore and Kannan (2022) extracted color, shape, and texture features using a discrete wavelet transform and adopted an AdaBoost SVM classifier, achieving an accuracy of 98.80%.
2. Rice disease identification based on convolutional neural networks (CNNs). With the widespread adoption of deep learning and outstanding performance of CNNs in image classification, CNN-based approaches have been increasingly applied to crop disease identification (Joseph et al., 2022). Liu et al. (2017) conducted apple leaf disease identification using an improved AlexNet model and achieved an accuracy of 97.62% on a self-constructed dataset, while enhancing the model’s robustness and generalization capability. Ferentinos (2018) evaluated five CNN architectures on the PlantVillage dataset, which included 25 crops and 58 disease categories. The experimental results showed that VGG achieved the best performance, with an accuracy of 99.53%. Rajasekaran et al. (2021) recognized 20 categories of tomato leaf diseases using an improved Xception model. For the dataset of 16,588 images combining PlantVillage and field-collected samples, the model achieved the highest accuracy of 99.55% using the Adam optimizer. Sharma and Kumar (2022) introduced an ensemble strategy based on transfer learning models for automated rice disease detection. This approach constructs different combinations using multiple pre-trained models such as InceptionV3, ResNet152V2, and MobileNetV2, and validates them through experiments on public datasets. The results indicate that most ensemble models outperform individual models, confirming the effectiveness of the ensemble method in improving the accuracy of rice disease recognition. Furthermore, with the rapid development of smart agriculture technologies and the widespread adoption of edge computing applications, the demand for lightweight network model architectures continues to grow. To achieve efficient and accurate rice disease recognition in resource-constrained field environments, a series of targeted lightweight models has been successively proposed. Zhang et al. (2025) proposed a lightweight and efficient network, LCE-Net, specifically designed for rice disease detection. The network backbone incorporates a scalable module called Scaling RepGhost-CSPELAN (SRG-CSPELAN), which enhances gradient flow and feature extraction capability while maintaining model compactness. Experimental results on both public rice disease datasets and a self-collected dataset show that LCE-Net outperforms several state-of-the-art methods in both accuracy and detection speed. On the public dataset, it achieves 95.0% accuracy with a detection time of 0.1901 seconds per image, while on the self-collected dataset, it achieves 98.6% accuracy at a speed of 0.0106 seconds per image. Wu et al. (2025) introduced a lightweight network based on filter sensitivity (FSLNet) for identifying rice leaf diseases. The core components of the algorithm include: a filter sensitivity evaluation algorithm (FSEval), a sensitive channel spatial attention mechanism (SCSAM), and a sensitivity-driven model compression method (SDMC). Experiments on a self-built dataset and the public Paddy Doctor dataset demonstrate that FSLNet, with only 0.38M parameters, achieves average accuracies of 95.26% and 97.98%, outperforming existing methods by 1.84% and 0.77%, respectively. Further tests on edge devices show that FSLNet achieves real-time high-precision recognition at a speed of 28 FPS. Lu et al. (2025a) proposed an Improved Mobile Transformer (IMobileTransformer) model for rice disease recognition. This model synergistically combines the advantages of MobileNet in local feature extraction and lightweight architecture with the superior ability of Transformer to handle global information. Experimental results on a self-built dataset show that, compared to classical models such as MobileNetV3-Large, EfficientNet-B0, ViT-B/16, and Swin-Transformer, the proposed model achieves a rice disease recognition accuracy of 99.62%, with an improvement of up to 38.09%, providing an efficient solution for rice disease identification.
3. Rice disease identification based on hybrid models combining CNNs and machine learning. Recent studies have explored the use of CNNs as feature extractors, followed by traditional machine learning classifiers, to build hybrid models that improve recognition performance (André et al., 2021). Feng et al. (2020) extracted high-dimensional features of rice leaf images using CNNs and combined them with an SVM for classification. Through ten-fold cross-validation, the model achieved an accuracy of 96.80%. Sethy et al. (2020) compared traditional feature extraction with CNN-based feature extraction on a dataset of 5,932 field images containing four rice disease categories. Their findings showed that a CNN + SVM hybrid model achieved an F1-score of 98.38%, significantly outperforming the handcrafted features. Chao et al. (2020) proposed the XDNet model, which integrates the Xception, DenseNet, and SVM structures. Through depthwise separable convolution and dense connections, the model enhanced the feature extraction capability and achieved an average accuracy of 98.35% on a self-constructed apple leaf disease dataset.

In summary, although deep learning has made significant progress in crop disease identification, several common challenges remain: First, general-purpose models often lack sufficient sensitivity to lesions with heterogeneous morphological features. Second, the robustness of model recognition under complex lighting conditions and varying backgrounds remains inadequate. Third, many models have large sizes and high computational complexity, making them difficult to deploy on resource-constrained mobile devices at the edge. Additionally, most existing research and practices focus on lowland rice, which grows in uniform environments and follows standardized data collection protocols. In contrast, the intelligent identification of diseases in mountain rice, the focus of this study, faces fundamental challenges stemming from its unique agroecosystem, making it an independent and more complex scientific issue. At the data acquisition level, fragmented and small-scale fields, cluttered image backgrounds, and the unpredictable mountain climate with frequent rain and fog result in unstable imaging conditions and inconsistent image quality. At the disease symptom level, nutrient stress due to poor soil often causes abnormal plant growth, whose phenotypes can easily be confused with disease symptoms, thereby increasing identification difficulty. At the model application level, inadequate network coverage in mountainous areas necessitates offline, low-power localized deployment solutions.

To address the aforementioned issues, this study proposed an edge-intelligent mountain rice disease identification model called LMRNet, which is based on CNNs. The architecture integrates multiple lightweight modules, including depthwise separable convolutions (Howard et al., 2017), grouped convolutions (Zhang et al., 2017), linear bottlenecks (Sandler et al., 2018), inverted residual blocks (Sandler et al., 2018), and channel shuffle mechanisms (Ma et al., 2018), to achieve both high efficiency and model compactness. Experimental results demonstrate that the proposed model can effectively identify rice diseases on the test dataset, exhibiting strong generalization capability and a small parameter size suitable for edge computing scenarios. Based on the proposed model, an Android-based mountain rice disease recognition application was further developed. Field validation experiments were conducted to evaluate the practicality and effectiveness of this application. The experimental results confirm the considerable potential for deployment in complex agricultural environments.

The main contributions of this study are as follows:

1. Design of a novel architecture: This paper proposes a novel convolutional neural network architecture named LMRNet, tailored for mountain rice leaf disease recognition. Its core innovation lies in constructing a lightweight feature extraction unit that deeply integrates group convolution, channel shuffling, and parallel downsampling. By leveraging structural reparameterization principles and multi-path feature fusion mechanisms, it effectively reduces computational complexity and memory consumption while preserving model representational capacity, thereby achieving an optimal balance between efficiency and robustness for edge deployment.
2. Application implementation: The newly proposed LMRNet architecture has been successfully deployed in an Android-based smartphone application, enabling real-time identification of mountain rice leaf diseases under offline conditions. This application enables agricultural practitioners to rapidly obtain disease information directly in the field, thereby reducing their reliance on onsite guidance from agricultural experts to a certain extent.
3. Model interpretability analysis: This study employed gradient-weighted class activation mapping (Grad-CAM) to visualize and analyze the feature extraction processes of the proposed LMRNet architecture and the comparison models. The analysis further revealed how the new architecture captures the semantic information of lesion regions within convolutional feature maps and how these features contribute to class discrimination. Moreover, in contrast to existing models, activation heatmap analysis showed that comparison models exhibiting signs of overfitting produce scattered attention regions, failing to effectively focus on typical lesion areas. This finding provides interpretability-based support for the quantitative experimental results, verifying the superior generalization ability of the proposed model and offering theoretical guidance for the future structural optimization of disease identification models.

The remainder of this paper is organized as follows. Section 2 provides a detailed description of the dataset used in this study, the design of the model architecture, the application development workflow, and the evaluation metrics for model performance. Section 3 presents the training conditions and parameter settings, followed by a systematic analysis of the experimental results, including performance comparisons with mainstream lightweight models and field validation experiments. Section 4 discusses interpretability analysis of the feature extraction process of the model, primarily focusing on the feature response regions when overfitting occurs. Finally, section 5 concludes the paper and outlines future research directions.

## 2 Materials and methods

### 2.1 Self-built dataset

#### 2.1.1 Data acquisition

This study referred to the *2024 Technical Plan for the Prevention and Control of Major Rice Diseases and Pests* issued by the China Agricultural Technology Extension Service Center and selected five major leaf diseases of mountain rice from the key disease list of the single-cropping rice production region in Southwest China as the research objects (Guo et al., 2025). Common diseases include rice blast, bacterial leaf blight, and sheath blight, whereas less common diseases include narrow brown spots and bacterial streak. The rice disease images were collected from April 2023 to September 2025 at the Three Gorges Comprehensive Experimental Station of the National Rice Industry Technology System, located at the Chongqing Academy of Agricultural Sciences. A smartphone was used as the image acquisition device, and its hardware specifications are listed in **Table 1**. The image acquisition conditions were randomized across multiple dimensions to ensure the validity and robustness of the dataset. The specific requirements were as follows: Lesion region: the main lesion area must remain intact with clearly defined boundaries; Illumination conditions: images were captured under various weather conditions, including sunny, cloudy, overcast, and postrain environments; Temporal conditions: the shooting time ranged from 05:00 to 19:00 within a day; Shooting distance: the distance between the smartphone and the target diseased leaf was maintained between 0.30 m and 0.50 m; and Shooting angle: the angle between the smartphone and the central axis of the lesion region was controlled within approximately ±30°. Using this diversified acquisition strategy, a field rice disease image dataset with substantial variability and complex backgrounds was constructed to provide a robust data foundation for the subsequent model training and evaluation.

**Table 1:** Data acquisition equipment parameters.

Camera Parameters	Huawei Mate 50	HONOR Magic5	Xiaomi 12 S	Apple 14
Pixel	50 million	50 million	50 million	48 million
Image resolution	8192 × 6144	8192 × 6144	8192 × 6144	8192 × 6144
Aperture	f/1.4	f/1.6	f/1.9	f/1.78
Sensor Model	IMX 766	IMX 700	IMX 707	IMX 703

During the field acquisition process, certain disease categories contained relatively few sample images, resulting in a class imbalance and insufficient sample diversity within the dataset. To mitigate this issue, this study further supplements the disease image samples through web crawling, with the retrieved data sourced from the Data Center of the Chinese Academy of Sciences (Chen et al., 2024), thereby enhancing the completeness and representativeness of the dataset and providing a more robust data foundation for model training and validation. By integrating onsite image acquisition with online image retrieval, a total of 3,256 rice disease images were collected. All images were saved in JPG format and uniformly processed into 448 × 448 resolution RGB images using Adobe Photoshop 2020 (version 21.0.1; Adobe Inc., San Jose, CA, USA).

#### 2.1.2 Data annotation

**Figure 1:**
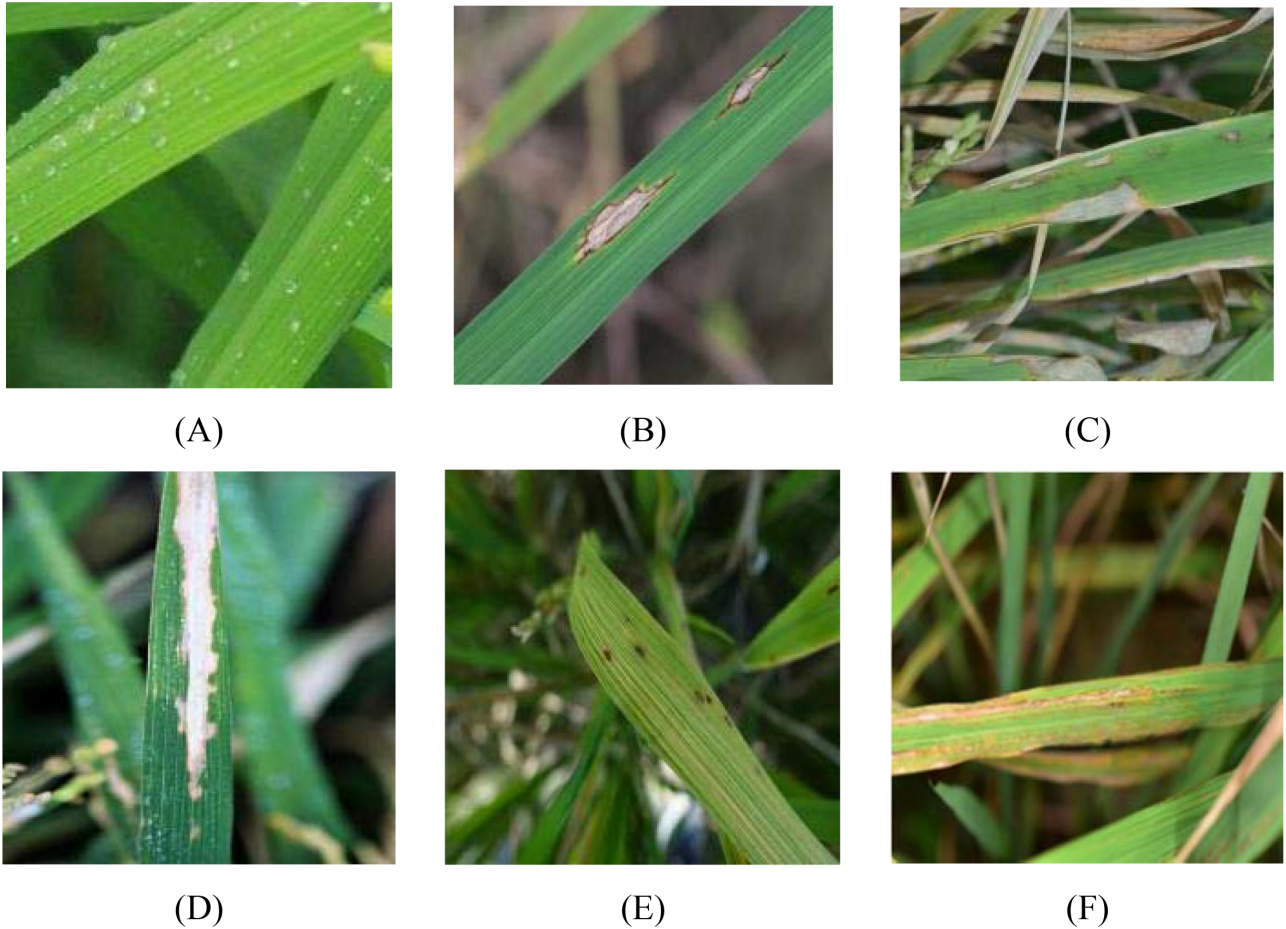
Examples of typical disease images. **(A)** Healthy; **(B)** Rice leaf blast disease; **(C)** Rice sheath blight; **(D)** Rice bacterial leaf blight; **(E)** Rice brown spot disease; and **(F)** Rice bacterial streak.

To account for inconsistencies in image quality caused by human factors and device variations, which may adversely affect the subsequent model training, a systematic data-cleaning procedure was conducted. The workflow included the following steps: 1) Manual preliminary screening: All images were inspected individually, and those of poor quality, such as blurred images, overexposed or underexposed lighting, severe occlusion, or incorrect labels, were removed. 2) Similarity detection: A pixel-based comparison algorithm was employed to identify highly similar images. Duplicate samples were manually examined to prevent redundant data from influencing the dataset. 3) Expert verification: With the assistance of plant protection experts, images exhibiting typical symptom characteristics for each disease category were finalized to construct a standardized dataset. The characteristic features of each disease and their corresponding manifestations are summarized in **Table 2**, and representative sample images are shown in **Figure 1**. Finally, 300 images were retained for each disease category, and 300 images of healthy rice leaves were preserved, resulting in 1,800 images for model training and testing.

**Table 2:** Typical disease characteristics and manifestations.

Serial number	Name	Lesion morphology	Lesion color	Lesion texture	Location of lesions
1	Healthy	Leaves are typically oblong in shape, with smooth, even margins free of visible damage or disease spots.	Healthy rice leaves typically exhibit a vibrant green color, with a uniform and bright hue.	The leaf surface is smooth and clean, free of water stains, mold, or slimy substances, and the leaves are dry.	−
2	Rice blast	The initial lesions appear as small, water-soaked or white, circular to irregular spots. As the disease progresses, these lesions gradually enlarge and coalesce into large gray-white or gray-green patches. The lesion margins are typically indistinct, and the affected area continues to expand with disease development.	The early lesions appear gray-white or gray-green, and as the pathogen produces spores in subsequent stages, the lesion color may darken to gray-black.	The lesion surface typically exhibits a water-soaked appearance, and in some cases, signs of tissue decay may be observed.	The lesions are primarily distributed on the leaves, particularly near the leaf tips or along the leaf margins.
3	Sheath blight	The early lesions appear as reddish-brown or dark brown elongated streaks, with serrated or wavy margins. As the disease progresses, the lesions gradually enlarge and merge, forming extensive longitudinal patches.	Initial lesions appear as reddish-brown or dark brown. As the disease progresses, the color of the lesions gradually darkens to dark brown or blackish-brown.	The surface of the lesions often exhibits minute depressions.	Lesions are primarily distributed on rice leaves, particularly at the leaf base and mid-section.
4	Bacterial leaf blight	Initially, the symptoms manifest as small yellowish-white spots. As the disease progresses, these spots gradually expand and coalesce into large yellowish-white lesions. The lesion margins are typically well-defined, exhibiting distinct annular or irregular shapes.	Initial lesions appear as small yellowish-white spots. As the disease progresses, the lesions gradually darken to yellowish-brown or dark brown.	Lesions typically appear water-soaked, and occasionally some rot may be observed.	Lesions are primarily distributed on rice leaves, particularly on the middle and lower parts of the leaves.
5	Rice brown spot	Initially appearing as small yellowish-brown spots, the lesions gradually enlarge and merge as the disease progresses, forming irregularly shaped yellowish-brown patches. The edges of the lesions often feature a black or dark-colored ring, clearly demarcating them from healthy tissue.	Initial lesions appear yellowish-brown. As the disease progresses, the lesions gradually darken, forming dark brown or blackish-brown patches.	The surface of the lesions often has a water-soaked appearance, and occasionally signs of rot may be observed.	Lesions are primarily distributed on rice leaves, particularly on the leaf surface.
6	Bacterial leaf streak	Initially appearing as elongated elliptical or linear lesions, these gradually enlarge as the disease progresses, forming elongated brown patches.	Initial lesions appear as reddish-brown or dark brown elongated streaks. As the disease progresses, the lesions gradually darken in color, turning brown or dark brown.	The surface of lesions often exhibits a water-soaked appearance, with relatively regular shapes. In severe infections, lesions may become sunken or develop areas of brown rot.	Lesions are primarily distributed on rice leaves, particularly at the leaf base and mid-section.

#### 2.1.3 Dataset partitioning

To ensure equitable data distribution and experimental reproducibility, the dataset was partitioned into a standard three-segment data structure (comprising training, validation, and test sets) in a 60:20:20 ratio (Babu et al., 2020). This partitioning strictly adhered to the principle of stratified sampling across all categories, and the validation set remained unseen during the training process. This dedicated dataset is designated as the Mountain Rice Leaf Disease Recognition Dataset (MRD-Leaf). The MRD-Leaf dataset comprises five classes of diseased rice leaf samples and one class of healthy leaf samples. The sample organization structure follows the Image Folder format, facilitating direct invocation and training within mainstream deep learning frameworks. The MRD-Leaf dataset contains several challenging “hard samples,” which significantly increase the complexity of the disease recognition task. These samples can be classified into two categories: (1) Small-object samples. These samples are represented by brown spots and feature minute lesion areas and inconspicuous visual features; and (2) Morphologically heterogeneous samples: These samples are exemplified by bacterial leaf streaks, the lesions exhibit a scattered morphological distribution, irregular margins, and complex color variations. The number of these challenging samples accounts for one-third of the total samples in the disease dataset, and their presence significantly increases the complexity of the disease recognition task. The specific partitioning details are listed in **Table 3**.

**Table 3:** Training, validation, and test set partitioning of the dataset.

Dataset category	Image dimensions (pixels)	Classification ratio (%)	Sample count (images)	Total samples (images)
Training set	512 × 512	60.00	1080	1800
Test set		20.00	360	
Validation set		20.00	360	

#### 2.1.4 Data preprocessing

##### 2.1.4.1 Data normalization

Agricultural field environments exhibit high randomness, and the occurrence of crop diseases presents significant uncertainty and heterogeneity, rendering data imbalances unavoidable (Lili et al., 2023). In contrast, CNN models are sensitive to the numerical distribution range of input features. If the feature values vary significantly, gradient oscillation may occur during model training, thereby affecting the convergence speed and final performance. To mitigate these issues and enhance the model training stability and generalization capability, this study employed the pixel normalization method (Sane and Agrawal, 2017). This technique performs mean subtraction and standard deviation normalization on each channel of the image.

##### 2.1.4.2 Data augmentation

CNNs possess a robust model generalization capability—their ability to accurately identify and classify unseen samples—which is one of the core advantages of image classification tasks. However, when the dataset size is limited and sample diversity is insufficient, the model is prone to overfitting during the training phase. This phenomenon is characterized by the excessive learning of the training samples, leading to inadequate generalization performance on the test set.

**Figure 2:**
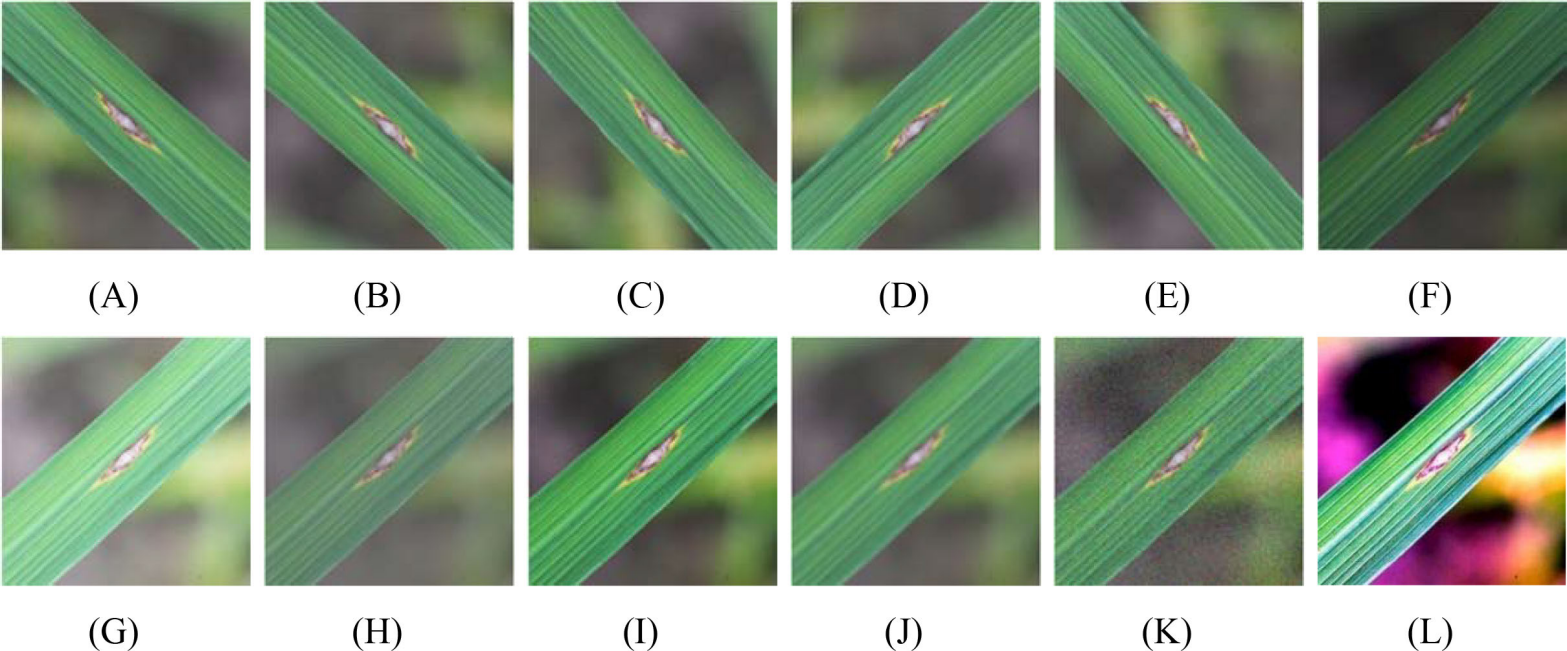
Visualization of data augmentation effects. **(A)** Horizontal mirror image; **(B)** Vertical mirror image; **(C)** Rotate 90°; **(D)** Rotate 180°; **(E)** Rotate 270°; **(F)** Low brightness; **(G)** High brightness; **(H)** Low contrast; **(I)** High contrast; **(J)** Gaussian noise; **(K)** Salt and pepper noise; **(L)** Color Perturbation.

To mitigate this issue and enhance the adaptability of the model to diverse samples encountered in real-world application scenarios, data augmentation techniques have become essential auxiliary tools. They serve not only to expand the sample size but also to boost variability and representativeness of the data. This study adopted the following data augmentation strategies (Mumuni and Mumuni, 2025): (1) Angular perturbation: Owing to constraints in real-world field acquisition, obtaining disease samples from multiple viewing angles is challenging. To enhance the model’s robustness against variations in input orientation, we employed angular rotation and horizontal/vertical mirroring transformation, effectively simulating multiview shooting scenarios; (2) Illumination perturbation: Acquisition time and meteorological conditions (e.g., sunny, overcast, and postrain) significantly affect image brightness and color balance, leading to variations in image quality. To simulate complex lighting changes, we applied brightness adjustments and contrast transformations to effectively mimic different illumination conditions; (3) Noise perturbation: Owing to inconsistencies in equipment stability and human operation, raw images are often accompanied by varying levels of noise that can interfere with model feature extraction. We introduced Gaussian noise and salt-and-pepper noise to effectively simulate image-corruption scenarios; (4) Color perturbation: A complex and variable field environment can easily cause color shifts in leaf images. To prevent the model from overfitting to the specific color characteristics of the training set, we utilized the principal component analysis color augmentation method to achieve a more realistic simulation of the color distribution. The specific augmentation effects are illustrated in **Figure 2**.

### 2.2 Proposed method

#### 2.2.1 Network model construction

This study proposed a lightweight CNN model, called the lightweight mountain rice leaf disease recognition network (LMRNet), specifically constructed for mountain rice leaf disease recognition. The LMRNet model demonstrates highly efficient operational performance and satisfactory feature extraction capability, which combinedly lay the foundation for an edge intelligence-oriented mountain rice leaf disease recognition system.

As listed in **Table 4**, the LMRNet model consists of a total of 8 stages. Stage 1 is a depthwise separable convolutional layer, composed of a depthwise convolution with a kernel size of 3 × 3 and a stride of 2, followed by a pointwise convolution with a kernel size of 1 × 1 and 24 filters. This layer serves as the initial feature extraction layer, aiming to initiate feature learning with minimal parameter overhead (approximately 8–9 times fewer parameters compared to standard convolution), thereby conserving memory and storage for subsequent computations. Stages 2 to 5 are feature extraction layers, each sequentially containing one stride-2 and one stride-1 basic feature extraction unit. This customized basic feature extraction unit, through the synergistic design of grouped convolution and channel shuffling, decomposes dense full-channel convolution operations into sparse, highly parallelizable grouped operations while maintaining strong representational capacity, significantly reducing multiply-accumulate operations (FLOPs) and serving as the key to achieving low-power inference on edge devices. Stage 6 is a feature channel adjustment layer, consisting of a pointwise convolution with a kernel size of 1 × 1 and 1000 filters. Stage 7 is a global average pooling layer with a pooling kernel size of 14 × 14 and 1000 pooling kernels, which avoids the massive parameter count associated with fully connected layers. The final stage is the Softmax classification layer.

**Table 4:** Key parameters of the LMRNet model.

Name	Output feature map	Convolution kernel size	Stride	Repetition count	Group count	Expansion coefficient	Output channels
Input	448 × 448	−	−	1	−	−	3
Stage 1	224 × 224	3 × 3	2	1	−	−	24
Stage 2	112 × 112	−	2	1	3	6	48
	112 × 112	−	1	1	3	6	48
Stage 3	56 × 56	−	2	1	3	6	96
	56 × 56	−	1	1	3	6	96
Stage 4	28 × 28	−	2	1	3	6	192
	28 × 28	−	1	1	3	6	192
Stage 5	14 × 14	−	2	1	3	6	384
	14 × 14	−	1	1	3	6	384
Stage 6	14 × 14	1 × 1	1	1	−	−	1000
Stage 7	1 × 1	14 × 14	1	1	−	−	1000
Classifier	1 × 1	−	1	1	−	−	k = 6

**Figure 3:**
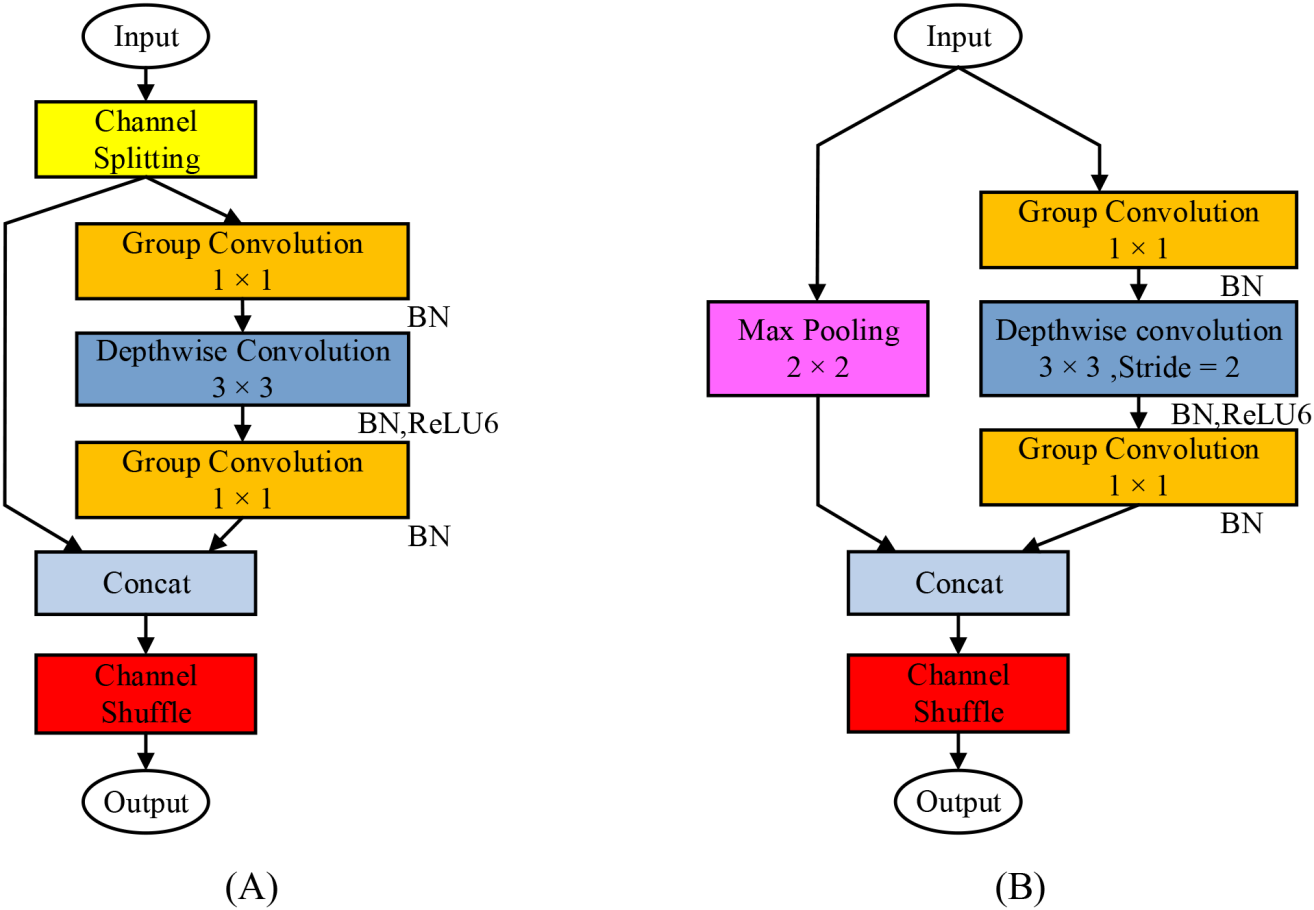
Basic feature extraction unit of the LMRNet model. **(A)** Step size of 1; **(B)** Step size of 2. This unit synergistically integrates an inverted residual structure, grouped convolution, and channel shuffling, effectively controlling computational complexity while preserving the richness of feature flow. It serves as the core module enabling the model to adapt to the computational constraints of edge devices.

The basic feature extraction unit of the LMRNet model is illustrated in **Figure 3**. In the stride-1 module, the intermediate feature map is passed into this extraction unit and first undergoes channel splitting, where the input feature map is divided into two parts with equal channel counts. The left branch uses identity mapping to directly propagate features, forming an inverted residual structure that alleviates gradient-related issues and avoids expensive full-channel convolution, significantly saving computational resources. The right branch sequentially performs the following operations: Channel Expansion: Grouped convolution (with group count g = 3and expansion factor t = 6), batch normalization (BN), and linear activation are applied, replacing full connectivity with grouped computation. This expands the feature dimension while reducing computational complexity by approximately a factor of g. Spatial Feature Extraction: Depthwise convolution (with a kernel size of 3×3and stride 1), BN, and ReLU6 activation are used to perform lightweight spatial filtering on the expanded channels, further extracting features with controlled computational overhead. Channel Compression: Grouped pointwise convolution (with group count g = 3), BN, and linear activation are applied to compress the feature map channels, adhering to the same low-overhead principle of grouped computation. Subsequently, the identity-mapped features from the left branch are concatenated channel-wise with the processed features from the right branch. The concatenated result then undergoes channel shuffling, which enables cross-group information exchange at minimal cost (without additional parameters), addressing the inherent “information isolation” issue of grouped convolution and ensuring feature quality. (Li et al., 2021) The stride-1 module maintains the spatial dimensions and channel count of the input and output feature maps, helping to enhance the model’s feature extraction capability.

In the stride-2 module, to achieve spatial downsampling of the feature map and channel expansion, the input feature map is fed into the feature extraction unit and first routed separately to the left maximum pooling branch and the right feature extraction branch. In the left maximum pooling branch, the feature map first passes through a max pooling layer with a kernel size of 2×2and a stride of 2. After max pooling, the spatial dimensions of the feature map are halved while the channel count remains unchanged. Based on the principle of max pooling (Ioffe and Szegedy, 2015), the core idea is to treat the element with the highest activation within a local window as the most representative feature signal. Since the feature responses output by convolutional layers are often non-uniformly distributed in space, max pooling extracts the most prominent response from each local window, effectively preserving the most discriminative local patterns in the image. For challenging samples such as rice bacterial leaf streak, which exhibit elongated and blurry-edged lesions, max pooling can capture a series of discrete yet highly responsive feature fragments along the direction of lesion extension. Although these fragments do not retain complete morphological continuity, they provide stable and critical discriminatory evidence for the classifier, thereby enhancing recognition robustness for elongated lesions while maintaining model lightweightness. In the second step, the feature map undergoes BN. BN normalizes the inputs of each batch so that their mean approaches 0 and variance approaches 1, thereby accelerating neural network training and improving model performance. In the right feature extraction branch, the stride of the depthwise convolution used for feature extraction is adjusted to 2, while other operations remain consistent with those in the stride-1 module. After feature extraction, the spatial dimensions of the feature map are halved, and the channel count remains unchanged.

Subsequently, the outputs of the two branches are channel-wise concatenated to achieve spatial downsampling and channel expansion, followed by channel shuffling. The stride-2 module provides nearly zero-computation-cost downsampling and salient feature retention through the max pooling pathway, combined with learnable downsampling via the convolutional pathway. Compared to using strided convolution alone, this design achieves a better balance between computational efficiency and feature richness while accomplishing spatial compression and channel expansion, directly contributing to low-latency inference of the model on edge devices.

#### 2.2.2 Application development

**Figure 4:**
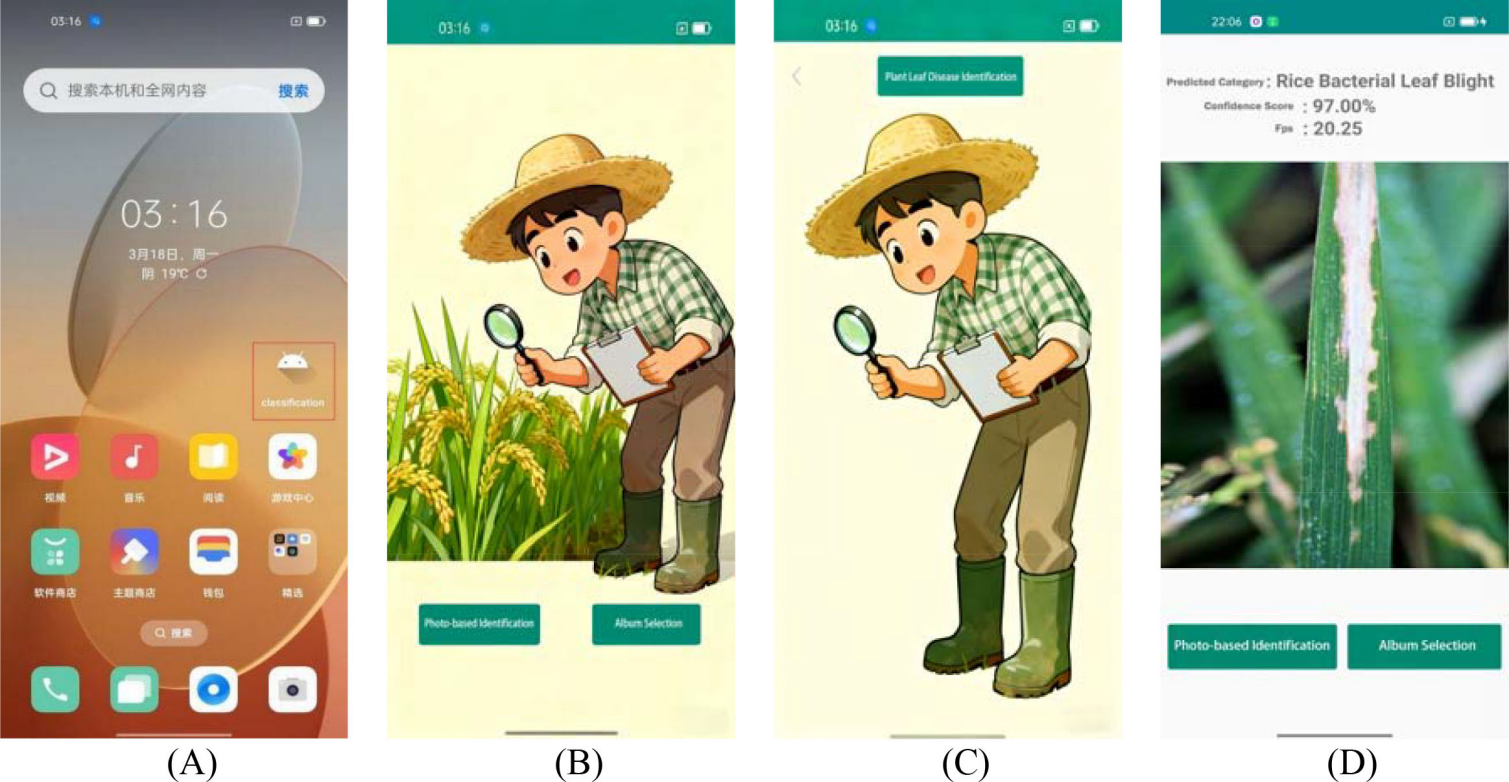
Mobile application user interface. **(A)** Application icon; **(B)** Start Screen; **(C)** Mode Selection; and **(D)** Recognition results.

To enhance the accessibility of the LMRNet model for agricultural practitioners, we developed a crop disease recognition application operating on an Android platform using Android Studio software (Version 21.3.1, Google Inc., CA, USA). The application interface is designed to be concise and intuitive, adhering to the philosophy of Integrated Operational Flow.” This design enables users to smoothly complete the entire process, from image acquisition to the presentation of recognition results. When using the application, users can either capture an image in real time via a smartphone camera or select an image from the local gallery. The system automatically initiates the analysis workflow upon loading the image and provides a real-time display of the disease recognition results. **Figure 4** illustrates the user interface of the developed application.

The LMRNet model is embedded within the application and runs locally on the device, eliminating the need for cloud-computing support. This design ensures high availability and response efficiency in remote areas with limited network coverage. Consequently, the application not only guarantees the immediacy and privacy of the prediction results but also reduces the resource overhead associated with data transmission, maintaining excellent adaptability and utility even in resource-constrained environments. Compared to the applications proposed by Manowarul et al (Manowarul et al., 2023), Zhang et al. (2020), and Ngugi et al. (2020), this application demonstrates superior real-time performance and accuracy. It provides farmers with immediate decision support, facilitates early disease intervention, and leads to enhanced crop health and increased yield.

### 2.3 Model performance evaluation metrics

In crop disease identification tasks, commonly used model performance evaluation metrics include classification accuracy metrics, such as accuracy, recall, precision, and F1-score. The model computational complexity metrics include floating-point operations (FLOPs), the number of parameters (params), and model size (Model Size). The model runtime efficiency is measured in frames per second (FPS) (Quan et al., 2024).

The various combinations arising from the comparison between the ground-truth image labels (true classes) and the predicted classes of the model can be categorized into four distinct outcomes: true positive (TP), false positive (FP), true negative (TN), and false negative (FN). The sum of these four outcomes is equal to the total number of samples evaluated.

Accuracy: This can be expressed as the proportion of correctly classified samples to the total number of samples in a given dataset, calculated using Equation 1:

(1) $$Accuracy=\frac{TP+TN}{TP+FP+TN+FN}$$

Recall: This can be defined as the proportion of correctly classified positive samples to the actual number of positive samples in a given dataset, calculated using Equation 2:

(2) $$Recall=\frac{TP}{TP+FN}$$

Precision: This can be defined as the proportion of correctly classified positive samples out of all samples predicted as positive within a given dataset. The calculation method is expressed in Equation 3.

(3) $$Precision=\frac{TP}{TP+FP}$$

F1 score: It can be expressed as the harmonic mean of precision and recall, combining the accuracy and completeness of the model. The calculation method is expressed in Equation 4.

(4) $$F1-score=\frac{2\times Precision\times Recall}{Precision+Recall}$$

Floating-point operations per second (FLOPs): This is used to measure model complexity.

Number of parameters (Params): This is used to measure model size.

Model Size (Model Size): Model Size refers to the storage space occupied by a trained neural network model.

FPS: This is used to measure computational efficiency.

## 3 Experiments and analysis

### 3.1 Model training

#### 3.1.1 Training conditions

(1) Hardware Configuration

Based on a thorough analysis of the experimental content in this study and the technical parameters of the core hardware required for training and optimizing CNN models, the hardware configuration parameters are listed in **Table 5**, considering factors such as operational efficiency, quality, and cost.

**Table 5:** Hardware configuration parameters.

Name	Configuration information
GPU	GeForce RTX 4090(24 G)
CPU	Intel(R) Xeon(R) Platinum 8352V CPU 2.10 GHZ
Memory	90 G
Hard Drive	300 G

(2) Software Environment

When conducting research on CNN models, deep learning frameworks are considered essential software tools that critically impact the quality and progress of model training and optimization. Based on the research content of this study and the requirements of the CNN models for software programming environments, the software environment configuration is presented in **Table 6**.

**Table 6:** Cloud software environment configuration.

Name	Configuration information
Operating Systems	Ubuntu 20.04
Programming Languages	Python 3.8.5
Deep Learning Frameworks	PyTorch 1.8.1
Scientific Computing Libraries	NumPy, SciPy, Scikit-learn, Pandas, Seaborn, Matplotlib
Computer Vision Libraries	OpenCV 4.8.0
Hardware Acceleration Libraries	CUDA 11.8, CUDNN 8.9.2
Development Environments	PyCharm Professional 2021.2

#### 3.1.2 Training parameters

The LMRNet model training parameters were selected based on factors such as dataset characteristics, network architecture, task type, hardware resources, and prior experience, as listed in **Table 7**.

**Table 7:** LMRNet model training parameter settings.

Name	Parameter information
Epoch	200 times
BatchSize	32
Optimizer	Stochastic Gradient Descent (SGD)
Learning Rate	0.01
Learning Rate Update Strategy	Fixed-Step Decay Learning Rate
Step Size	20
Gamma	0.5
Weight Decay	0.001
Loss Function	Cross Entropy Loss

#### 3.1.3 Training strategy

The LMRNet model employed a training approach that combined transfer learning and retraining. Phase One: First, the Plant Village dataset (Hughes and Salathe, 2015) was selected as the training sample for model pretraining. Subsequently, by freezing the pretrained weights of the bottom convolutional layers, the MRD-Leaf dataset was used as a training sample to update the parameters in the expansion layers of the model. This achieved the transfer of common image knowledge from the Plant Village dataset to a network model. Phase Two: Using the weights from phase one as the initial parameters, the model was retrained with the MRD-Leaf dataset to update all parameters. This strategy enabled the effective transfer of general knowledge learned from the Plant Village dataset, while allowing targeted fine-tuning and optimization of the entire network model for a specific task, thereby enhancing the overall performance.

### 3.2 Result analysis

#### 3.2.1 Model ablation study analysis

To accurately evaluate the contribution of each module in the LMRNet model to its overall performance, this study employs an ablation experiment methodology by progressively removing or replacing key modules to analyze their impact. The specific experimental designs are as follows: LMRNet−①: Replace all depthwise separable convolutions (or depthwise convolutions) in the model with standard convolutions. LMRNet−②: Replace all group convolutions with channel-wise convolutions. LMRNet−③: Remove the channel shuffle operation. LMRNet−④: Replace the max pooling operation in the stride-2 modules with standard convolutions. LMRNet−⑤: Remove the residual connections in the stride-1 modules. Under consistent training parameters and strategies, the original LMRNet serves as the baseline model for comparison. The results of the ablation experiments are presented in **Table 8**.

**Table 8:** Ablation experiment results.

Model name	Accuracy (%)	FLOPs (G)	Params (M)	Model size (M)	FPS
LMRNet(Baseline)	99.26	0.54	0.90	3.71	2066.31
LMRNet−①	98.52	1.20	2.50	10.30	945.68
LMRNet−②	98.43	1.05	2.10	8.65	1114.69
LMRNet−③	97.22	0.53	0.90	3.71	2110.12
LMRNet−④	98.70	0.65	1.10	4.53	1186.22
LMRNet−⑤	96.76	0.54	0.90	3.71	2084.36

①Depthwise separable convolution; ②Group convolution; ③Channel shuffle; ④Max pooling; ⑤Residual structure.

Based on the ablation study results presented in **Table 8**, the following observations can be made:

Replacing depthwise separable convolutions with standard convolutions (LMRNet-①) resulted in a substantial increase in computational cost (FLOPs) by 122% and parameter count (Params) by 178%, expanding the model size to 10.30 MB. Consequently, inference speed (FPS) dropped sharply by 54.2%. Despite the significant increase in model capacity, recognition accuracy (Accuracy) decreased by 0.74 percentage points. This indicates that depthwise separable convolution is not merely a lightweight alternative but a critical architectural design for maintaining the balance between high accuracy and efficiency in this network. Its efficient feature extraction capability cannot be equivalently replicated by standard convolutions.

Replacing group convolutions with channel-wise convolutions (LMRNet-②) also incurred significant efficiency costs, with FLOPs and Params increasing by 94% and 133%, respectively, accompanied by a corresponding increase in model size. Although inference speed decreased by 46.1%, accuracy declined by 0.83 percentage points—slightly more than in LMRNet-①. This further confirms that group convolution, while effectively reducing parameters, relies on its sparse connectivity pattern to preserve the model’s representational capacity, making it another essential pillar for achieving lightweight and high performance.

Removing the channel shuffle operation (LMRNet-③) had almost no impact on efficiency metrics (FLOPs, Params, Model Size), and inference speed slightly increased by 2.1% owing to operation simplification. However, recognition accuracy experienced the most severe drop, plummeting by 2.04 percentage points. This starkly demonstrates that channel shuffle acts as an information exchange hub in LMRNet. By facilitating cross-group channel communication, it effectively mitigates the “group isolation” of feature representations, making it a necessary condition for achieving high accuracy—its role cannot be compensated for by increasing computational complexity.

Replacing max pooling with a stride-2 convolution (LMRNet-④) introduced additional learnable parameters, increasing Params and Model Size by 22% and 22%, respectively, with FLOPs rising by 20.4%. Inference speed consequently decreased by 42.6%. However, this more flexible downsampling approach did not yield accuracy gains; instead, accuracy decreased by 0.56 percentage points. This suggests that the translation invariance and noise robustness of max pooling are more effective for the downsampling process in this task, whereas learnable convolutional downsampling may introduce unnecessary optimization challenges or overfitting risks.

Removing residual connections (LMRNet-⑤) had minimal impact on efficiency metrics and inference speed (slightly increased by 0.9%). However, accuracy suffered the largest drop of 2.50 percentage points. This clearly demonstrates that residual connections are a foundational structure ensuring effective training and stable performance in LMRNet. By alleviating gradient vanishing through identity mapping, residual connections enable the network to focus on learning residual features, thereby maintaining excellent performance even in deep models. Their contribution is structural and fundamental, and cannot be compensated for by adjusting other parameters.

The comprehensive ablation study results indicate that the high performance (high accuracy and efficiency) of the LMRNet network stems from the synergistic interaction of its components. Depthwise separable convolution and group convolution jointly form a lightweight yet expressive backbone for feature extraction. Channel shuffling serves as a critical information fusion mechanism, breaking inter-group isolation and unlocking the model’s representational potential. Max pooling provides robust and efficient downsampling, while residual connections ensure stable network optimization. Among these, channel shuffling and residual connections exert the most decisive influence on accuracy, whereas the lightweight convolution structures play a central role in balancing accuracy and efficiency. The absence or substitution of any single module disrupts this carefully designed equilibrium, leading to significant degradation in accuracy, efficiency, or both. This validates the rationality and necessity of the overall architectural design of LMRNet.

#### 3.2.2 Model comparison analysis

**Figure 5:**
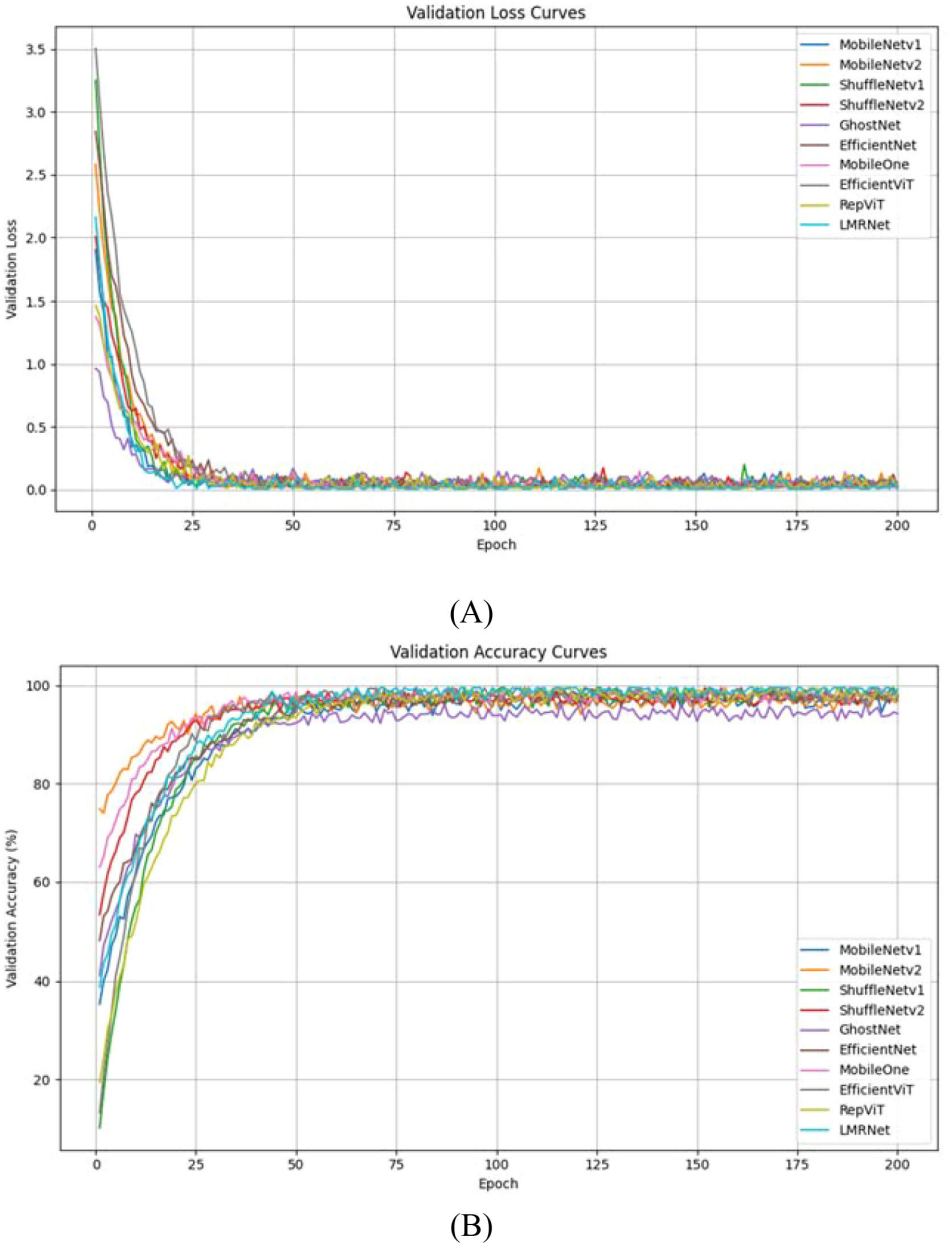
Visualization of model training loss curve and model validation loss curve. **(A)** Validation loss; **(B)** Validation accuracy.

**Figure 6:**
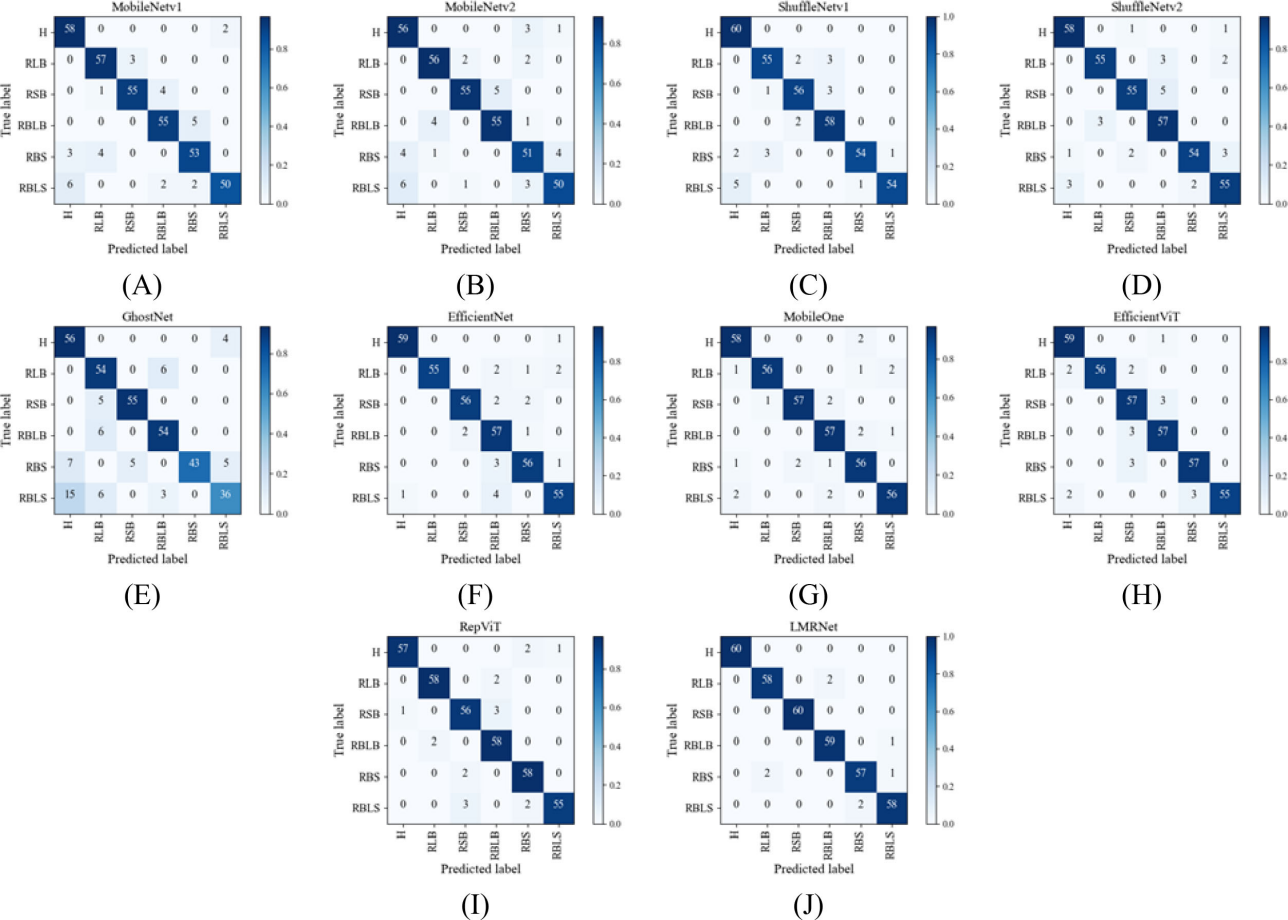
Confusion matrices of recognition results. **(A)** MobileNetv1; **(B)** MobileNetv2; **(C)** ShuffletNetv1; **(D)** ShuffletNetv2; **(E)** GhostNet; **(F)** EfficientNet; **(G)** MobileOne; **(H)** EfficientViT; **(I)** RepViT; **(J)** LMRNet H, Healthy; RLB, Rice Leaf Blast; RSB, Rice Sheath Blight; RBLB, Rice Bacterial Leaf Blight; RBS, Rice Brown Spot; RBLS, Rice Bacterial Leaf Streak.

The LMRNet model is compared with classic lightweight convolutional neural network models, including MobileNetv1 (Howard et al., 2017), MobileNetv2 (Sandler et al., 2018), ShuffleNetv1 (Zhang et al., 2017), ShuffleNetv2 (Ma et al., 2018), GhostNet (Han et al., 2020), EfficientNet (Tan and Le, 2019), MobileOne (Vasu et al., 2023), EfficientViT (Liu et al., 2023), and RepViT (Wang et al., 2024), under identical training parameters and strategies. The comparative experimental results of the obtained models are shown in **Table 9**, the visualization of the validation accuracy and validation loss curves is presented in **Figure 5**, and the confusion matrix of the model’s recognition results is displayed in **Figure 6**.

**Table 9:** Model performance comparison test results.

Model name	Accuracy(%)	Precision(%)	Recall(%)	F1-score(%)	FLOPs(G)	Params(M)	Model size (M)	FPS
MobileNetv1	97.04	91.33	91.11	91.09	2.31	3.22	13.05	1835.74
MobileNetv2	96.57	89.84	89.72	89.72	1.25	2.23	8.99	1182.45
ShuffletNetv1	97.87	93.84	93.61	93.61	0.57	0.91	3.78	1533.24
ShuffletNetv2	97.59	92.91	92.78	92.79	0.59	1.23	5.16	1326.33
GhostNet	94.26	84.21	82.78	82.52	0.58	2.68	10.96	1408.76
EfficientNet	97.96	94.21	93.89	93.96	2.94	4.02	16.48	1760.47
MobileOne	98.15	94.51	94.44	94.45	6.22	3.52	14.29	1381.58
EfficientViT	98.24	94.64	94.72	94.76	2.33	8.27	33.16	1420.32
RepViT	98.33	95.10	95.00	95.01	8.79	6.38	25.58	1408.12
LMRNet	99.26	97.78	97.78	97.78	0.54	0.90	3.71	2066.31

As listed in **Table 9**, the LMRNet model demonstrated superior performance compared with other classical lightweight CNN models in identifying leaf diseases in mountain rice. Specifically, in terms of classification metrics, LMRNet achieved an accuracy of 99.26%, a precision of 97.78%, a recall of 97.79%, and an F1-score of 97.78%. Regarding computational complexity metrics, LMRNet featured only 0.90 million parameters and 0.54 GFLOPs. In terms of runtime efficiency, it achieved 2066.31 FPS. All performance metrics demonstrated optimal levels.

From **Figure 5**, it can be observed that during the validation phase, the loss and accuracy curves of all models exhibit varying degrees of fluctuation. Specifically, the loss values tend to stabilize after approximately 25 training epochs, while the accuracy values gradually converge around 50 epochs. Comparing all models, the validation accuracy of GhostNet is significantly lower than that of the other models, indicating that GhostNet may suffer from a certain degree of overfitting.

From **Figure 6**, it can be observed that in terms of recognition performance across different disease categories, the LMRNet model demonstrates excellent results. In contrast, most classic lightweight convolutional neural network models, with the exception of RepViT, perform poorly in recognizing the two challenging sample categories: brown spot and bacterial leaf streak.

Comparative experiments demonstrated that the LMRNet model exhibited outstanding adaptability in identifying leaf diseases in mountain rice, achieving optimal recognition results. Consequently, this network model is suitable as a core algorithm for an Android-based smartphone application dedicated to identifying leaf diseases in mountain rice.

#### 3.2.3 Field validation test analysis

Although the LMRNet model constructed in this study achieved high recognition accuracy and computational efficiency, variations in recognition accuracy and operational efficiency owing to changes in input image quality and hardware computational capabilities must be considered when deployed in smartphone applications. To validate the performance of the application, three smartphones with distinct hardware configurations were selected for testing (see **Table 10** for specifications). Under identical experimental conditions, with a confidence threshold > 85% as the correct recognition threshold, validation tests were conducted using randomly captured images in the field. Thirty images were captured for each category, totaling 180 images, to assess the recognition accuracy and operational efficiency of the application. The detailed test results are presented in **Table 11**.

**Table 10:** Smartphone hardware specifications.

Mobile phone model	System	CPU	GPU	RAM	Camera
HONOR Magic VS	Android 14	Qualcomm Snapdragon8 Gen1	Adreno 730	16 GB	Sony IMX800
HONOR 400	Android 15	Qualcomm Snapdragon7 Gen4	Adreno 722	12 GB	Samsung HP3
OPPO A52	Android 11	Qualcomm Snapdragon 665	Adreno 610	8 GB	Unknown

**Table 11:** Field validation test results.

Disease Name	Mobile phone model	Number of disease samples (sheets)	Accurate identification of quantity (sheets)	Accuracy rate(%)	Average accuracy rate(%)	FPS	Average FPS
Health	HONOR Magic VS	30	29	96.67%	96.67	25.74	23.20
	HONOR 400	30	29	96.67%		23.21	
	OPPO A52	30	29	96.67%		20.65	
Rice Blast	HONOR Magic VS	30	29	96.67%	95.56	25.45	22.98
	HONOR 400	30	29	96.67%		22.68	
	OPPO A52	30	28	93.33%		20.83	
Sheath Blight	HONOR Magic VS	30	28	93.33%	91.11	25.67	23.19
	HONOR 400	30	27	90.00%		23.15	
	OPPO A52	30	27	90.00%		20.74	
Bacterial Blight of Rice	HONOR Magic VS	30	29	96.67%	96.67	25.17	23.41
	HONOR 400	30	29	96.67%		23.65	
	OPPO A52	30	29	96.67%		21.42	
Rice brown spot	HONOR Magic VS	30	26	86.67%	86.67	23.12	20.59
	HONOR 400	30	26	86.67%		20.10	
	OPPO A52	30	26	86.67%		18.56	
Bacterial Leaf Streak	HONOR Magic VS	30	26	86.67%	87.78	23.65	21.47
	HONOR 400	30	27	90.00%		21.20	
	OPPO A52	30	26	86.67%		19.56	

As listed in **Table 11**, sheath blight detection results were the most accurate, reaching 96.67% accuracy. This was followed by rice blast disease with an accuracy of 95.56%, whereas rice brown spot disease had the lowest accuracy of only 86.67%. The average recognition rate across all disease categories on the three smartphones was 92.41%, with an average recognition efficiency of 22.47 FPS. The validation experiments confirmed the high reliability of the system and demonstrated its capability to meet the expected goal of real-time leaf disease detection in mountain rice.

## 4 Discussion

The capability of CNN models to extract high-level semantic features from sample images is a key factor for distinguishing deep learning from traditional machine learning in the field of computer vision. After thoroughly validating the performance of the LMRNet model, we further explored how the model achieved feature understanding and feature distinction in the identification of foliar diseases in mountainous rice through convolutional operations.

### 4.1 Feature map heatmap analysis of the LMRNet model

**Figure 7:**
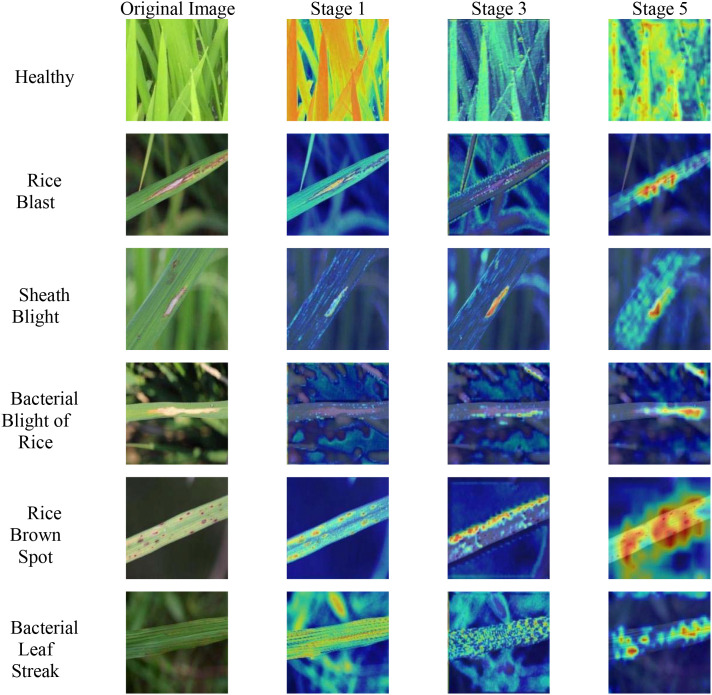
Heatmaps of feature mappings at various stages of the backbone feature extraction network of the LMRNet model.

With the assistance of the gradient-weighted class activation mapping method (Selvaraju et al., 2016), feature mapping heat maps at various stages of the lightweight backbone feature extraction network of the LMRNet model were obtained, as illustrated in **Figure 7**.

As shown in **Figure 7**, when the LMRNet model performs feature extraction on images of various disease categories, although the feature extraction process in CNNs involves weight sharing, the features extracted from the images of different disease categories are not identical.

The feature extraction process in CNNs can be divided into two stages: shallow and deep layers. The features extracted in the shallow layer primarily correspond to the local details and basic features of the image, such as edges, textures, and colors. This perfectly aligns with the highlighted regions in the feature map heatmaps of images from different disease categories in Stage 1. The LMRNet model primarily extracted color features from the leaf and lesion regions for rice blast disease (Magnaporthe oryzae) sample images, texture features from the leaf region and color features from the lesion region for sheath blight disease (Rhizoctonia solani) sample images, and texture features from the leaf region for bacterial blight (Xanthomonas oryzae) sample images. For rice brown spot disease (Alternaria sesami) sample images, the model mainly extracted color features from the leaf region and shape features from the lesion region. For the bacterial leaf streak disease (Xanthomonas vasicola) sample images, the color features were primarily extracted from the leaf and background regions. In contrast, because no distinct locally highlighted regions exist in healthy leaf sample images, the extracted features are global features.

Compared to the local details and basic features of shallow-layer features, deep-layer features are more abstract. They focused on the overall characteristics and semantic features of objects such as shape, structure, semantic information, contextual relationships, variations, and dynamic features. This is largely consistent with the information presented in the feature map heatmaps of images from different disease categories in Stage 5. Based on the architectural information of the LMRNet model, in Stage 5, the feature map size was downsampled to 14 × 14 pixels, meaning that each pixel in the current feature map represented 32 pixels of the original image. At this stage, the features are highly abstract, rendering it difficult to analyze the specific focus points of the network model. However, we can observe that the highlighted regions in the feature map heatmaps of disease sample images generally correspond to the main area of the lesion. According to the computational principle of Grad-CAM, the brightness of a feature map heatmap is proportional to the gradient value of the feature map. A higher gradient value indicates a greater influence of the feature map on the final classification result of the network model. Therefore, the features of the lesion regions in the sample images of diseases lay the foundation for the network model to make an accurate classification. Furthermore, the backbone feature extraction network of the LMRNet model can accurately locate the key regions of sample images and effectively capture critical and valuable feature information.

Furthermore, for healthy samples, the feature map heatmap exhibited nearly no concentrated regions of high or low brightness. Only small variations in brightness existed between the foreground and background regions. This further validates that the features of the lesion regions serve as the basis for accurate identification of the model. For diseases such as rice brown spot, which involve small target lesions, the feature map heatmap exhibits concentrated high-brightness regions that are primarily located in the individual lesion areas. However, other nonlesioned regions exhibit relatively high feature map values. This suggests that the CNN model incorporated a certain number of redundant features during the feature extraction process for rice brown spot disease. These redundant features are the main factors that contribute to inaccurate classification results. This theoretical conclusion aligns with the findings of the comparative experimental studies.

In the intermediate stage between the shallow and deep layers, specifically, Stage 3, the LMRNet model can focus on shallow features, assigning more learning weights to them and extracting deeper features. For instance, in the feature extraction of sheath blight disease sample images, the distribution of the highlighted regions in the feature map heatmap indicates that the LMRNet model still focuses on the texture features of the leaf region and the color features of the lesion region, but with an expanded focus and increased attention. Simultaneously, the model can transition from shallow to deeper features. For example, in the feature extraction of bacterial leaf streak disease sample images, the distribution of the highlighted regions in the feature map heatmap showed that the focus of the LMRNet model shifted from the more dispersed color features of the leaf and background regions to the more concentrated texture features of the leaf region, while primarily focusing on the shape features of the lesion region, thereby significantly increasing the feature map heatmap values of the lesion region.

### 4.2 Feature map heatmap analysis of the GhostNet model

**Figure 8:**
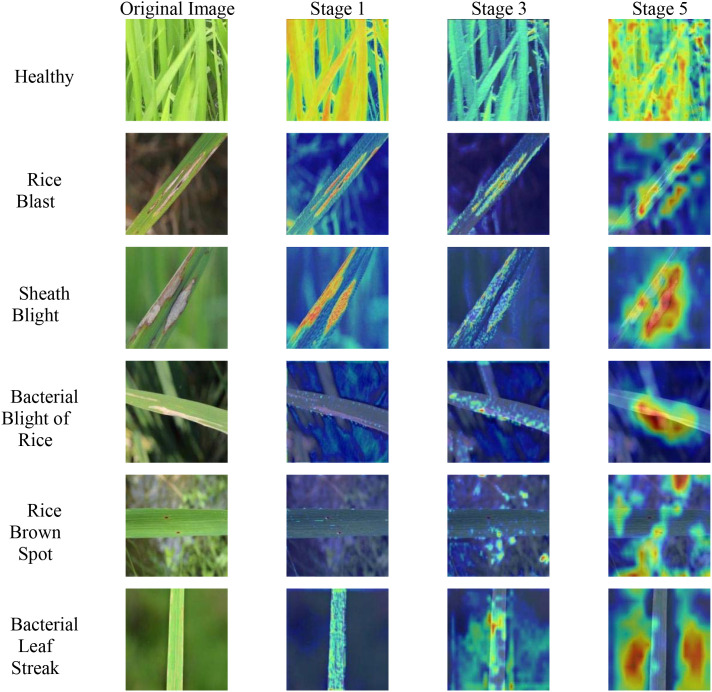
Feature map heatmaps at different stages of the backbone feature extraction network of the GhostNet model.

Compared to the existing studies by Lu et al. (2025b), Prashanthi et al. (2024), and Singh et al. (2023), this study not only focused on the feature extraction process of the self-built model but also emphasized the analysis of the feature extraction process of the comparison models. An analysis of the model training performance revealed that the GhostNet model exhibited a certain degree of overfitting. Therefore, it is hypothesized that the feature map heat map may present a form that is different from that of the LMRNet model. To demonstrate this difference, feature map heat maps at various stages of the backbone feature extraction network of the GhostNet model were obtained using Grad-CAM, as shown in **Figure 8**.

As shown in **Figure 8**, during the extraction of shallow and intermediate features, the backbone feature extraction network of the GhostNet model focuses on feature regions in a manner that is generally consistent with the LMRNet model. However, during deep feature extraction, for the three diseases with better recognition performances—rice blast, sheath blight, and bacterial blight—the feature focus areas of the backbone feature extraction network of the GhostNet model were also the lesion regions. In contrast, for the two diseases with poorer recognition performance, rice brown spot and bacterial leaf streak, the feature focus areas of the backbone network of the GhostNet model exhibited significant deviations. The high-brightness regions in the feature map heat map nearly completely avoided the lesion regions, and even the leaf region was excluded. At this point, the model relies on the background region as the primary basis for classification, leading to insufficient performance and generalization ability. This theoretical conclusion is consistent with the findings of previous comparative experimental studies.

With the assistance of the Grad-CAM method, we can observe that, during the CNN-based leaf recognition of mountain rice, the features of the lesion regions serve as the primary basis for correct disease identification by the CNN model. The ability of the model to extract features from lesion regions directly affects its performance.

### 4.3 Limitations

Although the LMRNet model proposed in this study demonstrates promising performance and practical deployment potential for mountain rice leaf disease recognition, several limitations remain:

Robustness in Extreme Environments:​ Despite incorporating diverse lighting conditions and viewing angles during data collection, the model’s recognition performance may degrade when faced with extreme in-field imaging conditions​ (e.g., severe backlighting, heavy occlusion, post-rain leaf glare). Systematic testing under such challenging conditions has not yet been conducted.

Insufficient Depth of Model Decision Interpretability:​ The current explanation for the model’s decision-making process relies primarily on qualitative observation. There is a lack of deeper analysis that integrates plant pathology knowledge​ to explain response biases in specific channels during feature fusion. Furthermore, quantitative analyses aimed at revealing the inherent mechanisms leading to misclassifications​ have not yet been performed.

Comprehensive Performance Evaluation for Edge Deployment:​ Although in-field validation tested average accuracy and frame rate, we have not yet systematically assessed the model’s long-term operational stability on mobile devices, power consumption profile, or its compatibility with low-end hardware.

## 5 Conclusion

This study proposed a lightweight convolutional neural network architecture, LMRNet, for the recognition of mountain rice leaf diseases. After conducting comparative experiments with typical lightweight models such as MobileNetv1, MobileNetv2, ShuffleNetv1, ShuffleNetv2, GhostNet, EfficientNet, MobileOne, EfficientViT, and RepViT, the results demonstrate that LMRNet outperforms these models in key evaluation metrics, including accuracy, precision, recall, and F1-score, on the MRD-Leaf dataset. Moreover, the model has a small number of parameters and a high computational efficiency, rendering it suitable for edge deployment in resource-constrained environments, particularly for real-time agricultural disease recognition tasks.

The mountain rice leaf disease recognition application developed in this study demonstrated good recognition accuracy and operational efficiency in offline multidisease recognition tasks across various models of Android smartphones. The application features a simple interface design and user-friendly interaction that can effectively assist agricultural practitioners in quickly obtaining disease information, improving field diagnosis efficiency, and reducing reliance on onsite guidance from experts, thereby providing technical support for disease monitoring and management.

Moreover, LMRNet exhibits good scalability and integration potential, providing foundational support for smart agriculture. Future research will further expand its functionality and applications in the following two directions: (1) Cross-Crop Generalization Validation: This study focuses on mountain rice disease recognition. In future work, the LMRNet model can be transferred to disease detection tasks for other important crops (such as wheat, corn, etc.) to validate its generalization capability and adaptability across different crop types and growing environments, thereby promoting the broader application of lightweight models in smart agriculture. (2) Model Deployment Performance Optimization: Future research will focus on conducting hardware-aware, comprehensive performance evaluations for real-world edge deployment scenarios. We will systematically measure key metrics of LMRNet—such as peak memory usage, model loading latency, and inference power consumption—on typical mobile devices. This will enable a thorough analysis of the model’s practical performance in resource-constrained environments, providing a reliable basis for lightweight deployment tailored to agricultural field applications, and ultimately enhancing the usability and practicality of the model in real-world production settings. (3) Development of a pesticide recommendation system: An intelligent pesticide recommendation module will be designed based on an MLP model and integrated with the disease recognition network. This system will enable a comprehensive analysis of disease types, occurrence times, and frequencies, providing optimal pesticide types and dosage recommendations, thus further advancing the development of precision agriculture and green pest control.

Overall, LMRNet not only demonstrates efficient and high-precision performance in mountain rice disease recognition tasks but also provides a feasible pathway for the deployability and sustainability of agricultural AI systems. It has a promising application potential and substantial value for widespread adoption.

## Data Availability

The raw data supporting the conclusions of this article will be made available by the authors, without undue reservation.

## References

[B1] André A. Afonso P. F. Flavio V. B. D. (2021). Plant diseases recognition on images using convolutional neural networks: A systematic review. Comput. Electron. Agric., 185. doi: 10.1016/J.COMPAG.2021.106125, PMID: 41842036

[B2] Babu D. Chaithanya M. Sandhya M. Shireesha G. (2020). Deep learning model for plant disease detection. Int. J. Recent Technol. Eng. 9, 750–754. doi: 10.35940/ijrte.A1232.059120

[B3] Chao X. Sun G. Zhao H. Li M. He D. (2020). Identification of apple tree leaf diseases based on deep learning models. Symmetry 12, 1065. doi: 10.3390/sym12071065, PMID: 41725453

[B4] Chen L. Zhou N. Zhu X. Yuan Y. (2024). Agricultural knowledge map construction data set. J. Agric. Big Data 6, 1–8. doi: 10.19788/j.issn.2096-6369.100002

[B5] Feng J. Yang L. Yu C. Di C. Li G. (2020). Image recognition of four rice leaf diseases based on deep learning and support vector machine. Comput. Electron. Agric. 179, 105824. doi: 10.1016/j.compag.2020.105824, PMID: 41842036

[B6] Ferentinos P. K. (2018). Deep learning models for plant disease detection and diagnosis. Comput. Electron. Agric., 145311–145318. doi: 10.1016/j.compag.2018.01.009, PMID: 41842036

[B7] Guo Y. Liu H. Zhuo F. Li Y. Zhu X. Ge Z. . (2025). Overview of the prevention and control of major crop diseases and insect pests in China in 2024. Chin. J. Plant Prot. 45(2), 69–72, 87.

[B8] Han K. Wang Y. Tian Q. Guo J. Xu C. Xu C. (2020). GhostNet: More Features From Cheap Operations. In: 2020 IEEE/CVF Conference on Computer Vision and Pattern Recognition (CVPR). Seattle, WA, USA: IEEE. 1577–1586. doi: 10.1109/CVPR42600.2020.00165, PMID:

[B9] Howard A. G. Zhu M. Chen B. . (2017). MobileNets: efficient convolutional neural networks for mobile vision applications. ArXiv. doi: 10.48550/arXiv.1704.04861, PMID: 41363103

[B10] Hughes D. P. Salathe M. (2015). An open access repository of images on plant health to enable the development of mobile disease diagnostics. Comput. Sci. doi: 10.48550/arXiv.1511.08060, PMID: 41363103

[B11] Ioffe S. Szegedy C. (2015). Batch normalization: accelerating deep network training by reducing internal covariate shift. CoRR. doi: 10.48550/arXiv.1502.03167, PMID: 41363103

[B12] Joseph S. D. Pawar M. P. Pramanik R. (2022). Intelligent plant disease diagnosis using convolutional neural network: a review. Multimedia Tools Appl. 82, 21415–21481. doi: 10.1007/s11042-022-14004-6, PMID: 41841152

[B13] Kalita B. Kumar J. C. Hazarika N. Baruah K. K. Borah L. (2024). Exploring climate change adaptation practices and agricultural livelihoods among rice farmers of the brahmaputra valley in Northeast India. Environ. Manage. 73, 1180–1200. doi: 10.1007/s00267-024-01954-w, PMID: 38489036

[B14] Kishore K. K. Kannan E. (2022). Detection of rice plant disease using AdaBoostSVM classifier. Agron. J. 114, 2213–2229. doi: 10.1002/agj2.21070, PMID: 41837764

[B15] Li G. Zhang M. Li J. Lv F. Tong G. (2021). Efficient densely connected convolutional neural networks. Pattern Recognit. 109, 107610. doi: 10.1016/j.patcog.2020.107610, PMID: 41842036

[B16] Lili L. Bin W. Yanwen L. Hua Y. (2023). Diagnosis and mobile application of apple leaf disease degree based on a small-sample dataset. Plants 12(4), 786. doi: 10.3390/plants12040786, PMID: 36840133 PMC9964512

[B17] Liu X. Peng H. Zheng N. (2023). EfficientViT: Memory Efficient Vision Transformer with Cascaded Group Attention. arXiv [Preprint]. abs/2305.07027. doi: 10.48550/arXiv.2305.07027, PMID: 41363103

[B18] Liu B. Zhang Y. He D. Li Y. (2017). Identification of apple leaf diseases based on deep convolutional neural networks. Symmetry 10(1), 11. doi: 10.3390/sym10010011, PMID: 41725453

[B19] Lu F. Shangguan H. Yuan Y. Yan Z. Yuan T. Yang Y. . (2025b). LeafConvNeXt: Enhancing plant disease classification for the future of unmanned farming. Comput. Electron. Agric. 233, 110165. doi: 10.1016/j.compag.2025.110165, PMID: 41842036

[B20] Lu Y. Zhou H. Wang P. Wang E. Li G. Yu T. . (2025a). IMobileTransformer: A fusion-based lightweight model for rice disease identification. Eng. Appl. Artif. Intell. 161, 112271. doi: 10.1016/j.engappai.2025.112271, PMID: 41842036

[B21] Ma N. Zhang X. Zheng H. Sun J. (2018). ShuffleNet V2: practical guidelines for efficient CNN architecture design. arXiv [Preprint]. arXiv:1807.11164. doi: 10.1007/978-3-030-01264-9_8, PMID: 41841130

[B22] Madhavan V. M. Thanh H. N. D. Khamparia A. Pande S. Malik R. Gupta D. (2021). Recognition and classification of pomegranate leaves diseases by image processing and machine learning techniques. Comput. Mater. Continua 66(3), 2939–2955. doi: 10.32604/cmc.2021.012466, PMID: 40612875

[B23] Manowarul M. I. Ahad A. M. A. Alamin M. T. Uddin K. M. A. Ashraf M. U. Kamran M. H. . (2023). DeepCrop: Deep learning-based crop disease prediction with web application. J. Agric. Food Res. 14, 100764. doi: 10.1016/J.JAFR.2023.100764, PMID: 41842036

[B24] Mumuni A. Mumuni F. (2025). Data augmentation with automated machine learning: approaches and performance comparison with classical data augmentation methods. Knowledge Inf. Syst. 67, 1–51. doi: 10.1007/s10115-025-02349-x, PMID: 41841152

[B25] Nagpal J. Goel L. (2025). ResdenseNet: a lightweight dense ResNet enhanced with depthwise separable convolutions and its applications for early plant disease classification. Neural Computing Appl. 37, 1–22. doi: 10.1007/s00521-024-10972-y, PMID: 41841152

[B26] Ngugi L. C. Abdelwahab M. Abo-Zahhad M. (2020). Tomato leaf segmentation algorithms for mobile phone applications using deep learning. Comput. Electron. Agric. 178, 105788. doi: 10.1016/j.compag.2020.105788, PMID: 41842036

[B27] Pandi S. S. Gitanjali J. Pounambal M. ArivuSelvan K. (2023). Optimizing rice plant disease detection with crossover boosted artificial hummingbird algorithm based AX-RetinaNet. Environ. Monit. Assess. 195(9), 1070. doi: 10.1007/s10661-023-11612-z, PMID: 37610473

[B28] Phadikar S. Sil J. Das K. A. (2013). Rice diseases classification using feature selection and rule generation techniques. Comput. Electron. Agric., 9076–9085. doi: 10.1016/j.compag.2012.11.001, PMID: 41842036

[B29] Prashanthi B. Krishna P. V. A. Rao M. C. (2024). LEViT- Leaf Disease identification and classification using an enhanced Vision transformers (ViT) model. Multimedia Tools Appl. 84(21), 1–32. doi: 10.1007/s11042-024-19866-6, PMID: 41841152

[B30] Quan S. Wang J. Jia Z. Xu Q. Yang M. (2024). Real-time field disease identification based on a lightweight model. Comput. Electron. Agric. 226, 109467. doi: 10.1016/j.compag.2024.109467, PMID: 41842036

[B31] Rajasekaran T. Anandamurugan S. Kaliappan V. K. (2021). Automated tomato leaf disease classification using transfer learning-based deep convolution neural network. J. Plant Dis. Prot. 128, 73–86. doi: 10.1007/s41348-020-00403-0, PMID: 41841152

[B32] Sandler M. Howard A. Zhu M. Zhmoginov A. Chen C. L. (2018). MobileNetV2: Inverted Residuals and Linear Bottlenecks. In: 2018 IEEE/CVF Conference on Computer Vision and Pattern Recognition (CVPR). Salt Lake City, UT, USA: IEEE. 4510–4520. doi: 10.1109/CVPR.2018.00474, PMID:

[B33] Sane P. Agrawal R. (2017). Pixel normalization from numeric data as input to neural networks. Department of Computer Engineering SIES GST, University of Mumbai Mumbai, India;Department of Computer Engineering SIES GST, University of Mumbai Mumbai, India.

[B34] Selvaraju R. R. Cogswell M. Das A. Vedantam R. Parikh D. Batra D. (2016). Grad-CAM: Why did you say that? Visual Explanations from Deep Networks via Gradient-based Localization. arXiv [Preprint]. arXiv:1610.02391. doi: 10.48550/arXiv.1610.02391, PMID: 41363103

[B35] Sethy P. K. Barpanda N. K. Rath A. K. Behera S. K. (2020). Deep feature based rice leaf disease identification using support vector machine. Comput. Electron. Agric. 175, 105527. doi: 10.1016/j.compag.2020.105527, PMID: 41842036

[B36] Sharma M. Kumar J. C. (2022). Improving rice disease diagnosis using ensemble transfer learning techniques. Int. J. On Artif. Intell. Tools 31. doi: 10.1142/S0218213022500403, PMID: 40951326

[B37] Sharma M. Kumar J. C. Bhattacharyya K. D. (2024). Machine/deep learning techniques for disease and nutrient deficiency disorder diagnosis in rice crops: A systematic review. Biosyst. Eng. 244, 77–92. doi: 10.1016/j.biosystemseng.2024.05.014, PMID: 41842036

[B38] Sharma M. Kumar J. C. Singh P. T. Talukdar J. Saikia R. K. Gogoi A. . (2023). Enhancing disease region segmentation in rice leaves using modified deep learning architectures. Arch. Phytopathol. Plant Prot. 56(20), 1555–1580. doi: 10.1080/03235408.2024.2310326, PMID: 41799851

[B39] Shrivastava V. K. Pradhan M. K. (2021). Rice plant disease classification using color features: a machine learning paradigm. J. Plant Pathol. 103(1), 17–26. doi: 10.1007/s42161-020-00683-3, PMID: 41841152

[B40] Singh P. T. Shubhangi C. Pritee K. Tanuja S. Aparajita O. (2023). Vision transformer meets convolutional neural network for plant disease classification. Ecol. Inform. 77, 102245. doi: 10.1016/j.ecoinf.2023.102245, PMID: 41842036

[B41] Tan M. Le Q. (2019). EfficientNet: Rethinking Model Scaling for Convolutional Neural Networks. arXiv [Preprint]. arXiv:1905.11946. doi: 10.48550/arXiv.1905.11946, PMID: 41363103

[B42] Upadhyay A. Chandel N. S. Singh P. K. Chakraborty S. K. Nandede M. B. Kumar M. . (2025). Deep learning and computer vision in plant disease detection: a comprehensive review of techniques, models, and trends in precision agriculture. Artif. Intell. Rev. 58(3), 92. doi: 10.1007/s10462-024-11100-x, PMID: 41841152

[B43] Vasu A. K. R. Gabriel J. Zhu J. Tuzel O. Ranjan A. (2023). MobileOne: An Improved One millisecond Mobile Backbone. In: 2023 IEEE/CVF Conference on Computer Vision and Pattern Recognition (CVPR). Vancouver, BC, Canada: IEEE. 7907–7917. doi: 10.1109/CVPR52729.2023.00764, PMID:

[B44] Wang A. Chen H. Lin Z. Han J. Ding G. (2024). RepViT: Revisiting Mobile CNN From ViT Perspective. In: 2024 IEEE/CVF Conference on Computer Vision and Pattern Recognition (CVPR). Seattle, WA, USA: IEEE. 15909–15920. doi: 10.1109/CVPR52733.2024.01506, PMID:

[B45] Wu Y. Long W. Liu H. Wang H. Li S. Zhu R. . (2025). FSLNet: Filter sensitivity-based lightweight network for rice leaf disease recognition. Comput. Electron. Agric. 237, 110751. doi: 10.1016/j.compag.2025.110751, PMID: 41842036

[B46] Zhang Y. Guo R. Li M. (2025). LCE-Net: an efficient network for rice disease detection based on lightweight convolution. Measurement Sci. Technol. 36, 115404–115404. doi: 10.1088/1361-6501/ae1a07, PMID: 41725453

[B47] Zhang S. Huang W. Wang H. (2020). Crop disease monitoring and recognizing system by soft computing and image processing models. Multimedia Tools Appl. 79, 1–12. doi: 10.1007/s11042-020-09577-z, PMID: 41841152

[B48] Zhang X. Zhou X. Lin M. Sun J. (2017). ShuffleNet: An Extremely Efficient Convolutional Neural Network for Mobile Devices. arXiv [Preprint]. arXiv:1707.01083. doi: 10.48550/arXiv.1707.01083, PMID: 41363103

